# Caveats for the Good and Bad of Dietary Red Meat

**DOI:** 10.3390/antiox8110544

**Published:** 2019-11-12

**Authors:** Anthony T. Omaye, Stanley T. Omaye

**Affiliations:** Department of Nutrition, University of Nevada, Reno, NE 89557, USA

**Keywords:** heme iron, oxidative stress, antioxidants, fruits and vegetables

## Abstract

Red meat and its constituents of heme iron or free iron have been the target of scrutiny related to their purported association to many chronic diseases. However, in contrast, red meat provides a rich source of nutrition. In 2007, Al Tappel hypothesized that the mechanistic explanation for the adverse impact of iron and heme iron could be the strong influence these substances have in initiating and promoting oxidative stress. Also, there is an emphasis on the importance of dietary antioxidants in the modulation of these adverse effects. The goal of this argumentative review is to provide an update of the importance of dietary red meat for health, and the hypothesis that oxidative stress initiated by dietary iron and heme iron may be related to chronic diseases, with a particular emphasis on recent research that impacts the paradigm. We also examine potential dietary changes that could substantially modify the potential adverse outcomes of chronic diseases initiated by heme iron mechanisms, e.g., consumption of antioxidant-rich fruits and vegetables.

## 1. Introduction

Dietary meat has been an essential staple of human diets and has evolved over eras. There are those who assert it would be unlikely that meat, as such, could lead to many purported unhealthy conditions [1,2,3]. Dietary red meat is an excellent source of minerals and vitamins, mainly; zinc, iron, selenium, and vitamin B12 [4]. It is one of the primary sources of complete protein and energy in many countries globally [5,6]. It is important to note that treatable anemia, related to iron deficiency, is a critical global issue [7], and dietary meat consumption is preventative. Reduction of red meat consumption by women in Australia has been associated with lower levels of iron in their diets, which lead to higher rates of anemia yet treatable with diets containing meats. 

Although the direct association of fat content and quality of fat content of meats likely contribute to health issues, considerable concerns have been expressed regarding the heme iron in red meats as being responsible for the initial steps leading to many chronic diseases, e.g., cancers, heart disease, and neurologic abnormalities [8]. The focal point of these concerns is that heme iron can be a catalyst for oxidative stress and that oxidative stress can be an initiating step to chronic diseases. 

In this argumentative literature review, we discuss the importance of dietary red meat for health and the hypothesis that oxidative stress initiated by dietary iron and heme iron may be related to chronic diseases, with particular emphasis on recent research that impacts the paradigm. Also, we explore potential dietary changes that could substantially modify the potential adverse outcomes of chronic diseases initiated by heme iron mechanisms. For our searches, were used the University of Nevada (UNR) library “One Search,” which searches 600 million books, e-books, scholarly articles. It includes: items physically located at the UNR libraries and research databases, E-journal articles, e-books, streaming media, and other subscription content. Criteria, terms, or phrases used in our searches included: red meat or processed meat, iron, and heme iron, oxidation, lipid peroxidation, and antioxidant.

## 2. Iron, Heme and Oxidative Injury

Over ten years ago, the late Al Tappel hypothesized that the heme iron of red meat is likely a catalyst for oxidative damage promoting many chronic diseases [8]. Red meat contains oxymyoglobin, deoxymyoglobin, oxyhemoglobin, deoxyhemoglobin, cytochromes of mitochondria, and other heme pigments. After ingestion, heme proteins are hydrolyzed to amino acids and peptides and the heme group. The iron of the heme group can coordinate with potent ligands, such as sulfur, nitrogen, or oxygen of amino acids and other constituents. 

Iron is an indispensable element for almost all organisms; however, in excess, iron can be toxic, promoting redox reactivity and oxidative stress. Balanced iron metabolism is the key to health and preventing associated iron diseases. Free iron and coordinated heme are readily absorbed and transported by the body to organ/cellular components, and because of tightly controlled mechanisms, iron is unavailable to initiate oxidation. Usually, the organism contains heme compounds in protective cells or subcellular locations, for example, as hemoglobin in red blood cells or the confines of the spleen. Alternatively, iron is located in subcellular locations, like mitochondria, for the cytochromes of the electron transport chain. Most of the body iron in animals is distributed in red blood cells (>70%), which carries oxygen throughout the body. Macrophages remove senescent red blood cells and recycle iron to erythroblasts for utilization. The iron that is released into plasma is mediated by ferroportin (ferrous iron exporter). Subsequently ferrous iron undergoes oxidation to ferric ion by ceruloplasmin (ferroxidase) for iron carrier transferrin, which keeps iron in a redox neutral state. Transferrin delivers iron to tissue transferrin receptor 1 (TR1). The iron regulatory hormone, hepcidin, controls iron entry into the blood by binding to ferroportin in tissue macrophages, gut enterocytes, and other cells; therefore, resulting in sequestering iron within macrophages, inhibiting gut iron absorption and a drop in plasma iron levels. This reaction is a physiological response to inflammation whereby hepcidin induction serves to promote hypoferremia, sequestration in macrophages to deprive invading bacteria of essential iron. Prevention of iron access to pathogenic bacteria is an essential immune response of the body, often complemented by a variety of proteins that sequester iron, such as lactoferrin, transferrin, and other iron-binding proteins [9]. 

Hemochromatosis is a genetically heterogeneous endocrine disease of iron overload, which varies in molecular etiology and clinical manifestations. Since the iron sequestering of such proteins is compromised, hemochromatosis patients can be vulnerable to infections that typically do not affect other healthy individuals. Usually, there is disruption of the hepcidin pathway producing insufficient hepcidin responses to iron intake or too high body iron stores and subsequent loss of hepcidin-mediated feedback inhibition of dietary absorption, whereby the extent of hepcidin inhibition is correlated with the degree of systemic iron overload. The consequences of excess iron would be redox changes and oxidative stress. Untreated hemochromatosis can lead to multi-organ damage and failure—also, liver fibrosis, cancer, diabetes mellitus, osteoporosis, cardiomyopathy, and hypogonadism. Free and heme iron (coordinated iron) catalyze oxidation reactions. The Haber-Weiss reaction generates hydroxyl radicals for hydrogen peroxide, which can be a source of oxidative stress in cells. The first stage of the catalytic cycle is the reduction of ferric iron to ferrous ion.

Fe^3+^ + O_2_^−^ → Fe^2+^ + O_2_

The following step, known as the Fenton reaction:

Fe^2+^ + H_2_O_2_ → Fe^3+^ + OH^−^ + OH•

**Figure 1:**
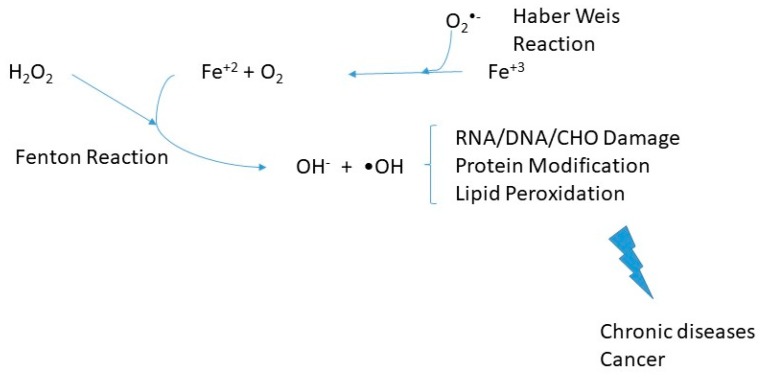
Oxidative stress-induced by iron metabolism derangements. Products of oxidative stress can result in ribonucleic acid (RNA), deoxyribonucleic acid (DNA), and carbohydrate (CHO) damage and protein modification/lipid peroxidation. Such changes can eventually lead to chronic diseases and cancer [10].

Such reactions, illustrated in Figure 1, likely are a prelude to lipid peroxidation or subsequent chain reactions and oxidative stress in cells and organs. Oxidative stress can result in damage to biological molecules, including DNA (in a prelude to cancers) and other diseases. Heme catalyzed oxidations are the strongest oxidizing system for developing tissue damage. 

## 3. Meat, Iron, and Food Processing

Red meat consumption has been related to cancers, particularly colon and rectal cancers. The endogenous formation of intestinal carcinogenic N-nitroso compounds [11,12,13] are contributing substances. It has been suggested that this effect is mediated by heme iron. Also, heme iron produces N-nitroso compounds in processed meat, and there are other compounds produced by cooking, such as heterocyclic amines (HCAs) and polycyclic aromatic hydrocarbons (PAH). 

Nitrates are relatively inert unless converted to nitrites, which can occur by bacteria, present in plants as well as animals; therefore, naturally in food. Conversion from nitrate to nitrite can occur in saliva due to bacteria. The purpose of adding nitrites is to prevent harmful bacteria in meats and giving the pink or red color to meats. Nitrites can be converted into nitric oxide, which reacts with the oxygen-binding proteins in the meat, changing its color. Nitrite plays a significant role in inhibiting the growth of foodborne pathogens, including *Clostridium botulinum* (*C. botulinum*) that causes botulism, a life-threatening disease. However, *N*-nitroso compounds can be created from nitrite, which is considered carcinogenic. Nitrate is added in meat at low levels, and it is improbable that such levels would produce deleterious health conditions. Although more research is warranted, considerable negative publicity has influenced some food manufacturers in not using nitrate in their food processing products.

HCAs formed from cooking meats have been suggested to be related to cancers and have been demonstrated in animals subjected to very high concentrations [14]. HCAs are formed when proteins, sugars, and creatine react at high temperatures. Modulation of the adverse effects is likely to be a function of cooking at high temperatures or long duration, i.e., reduced to lower temperatures at shorter cooking times. Also, subsequent removal of juices produced by precooking (heating in the microwave for short duration) can be a useful technique to lower HCAs. Moreover, epidemiological studies failed to find a relationship between higher intakes of heterocyclic amines by people who had cancer compared to healthy populations [15,16]. High-temperature cooking can contribute to high levels of advanced glycation end products (AGEs), which have been shown to increase oxidative and inflammatory reactions [17,18,19].

In epidemiology studies, augmented risks have been detected with unhealthy eating and larger portion sizes of well-done or charred meat cooked at excessive temperatures. PAHs occur when fat and juices from meat are prepared directly above an open fire and drip onto the fire, causing flames [20,21,22]. These flames have PAHs that then stick to the surface of the meat. PAHs can also occur during other food preparation processes, such as smoking of meats. Iron in red meats has been studied extensively to determine its contribution to colon cancer development. As noted above, iron is potentially mutagenic due to the formation of hydroxyl radicals and other byproducts that suppress the activity of host defense cells and promote mutagenicity. Heating temperature, likewise, is directly related to oxidation and potential mutagenicity of meat and meat products. 

## 4. Population Studies with Correlations between Red Meat Consumption and Chronic Diseases

Over the previous few decades, one can find many citations of epidemiological correlations and mechanistic studies suggesting red meat correlations and heme iron as causative for chronic diseases. In some studies, stronger positive associations were found between either red meat intake or heme iron intake and colorectal cancer risk [23,24,25,26,27] and cancers of other organs or tissues [28]. Also, correlations have been found between red meat and heart disease, rheumatoid arthritis, and type 2 diabetes [29,30,31]. In a 2015 report, 22 scientists from ten countries took the position that it is prudent to limit red meats in human diets. They classified consumption of processed meat as “carcinogenic to humans” (Group 1–there is enough evidence to conclude that it can cause cancer in humans) and consumption of red meat as “probably carcinogenic to humans” (Group 2A–there is strong evidence that it can cause cancer in humans, but at present it is not conclusive) [32]. However, this position has not been met with science-based consensus. In contrast, others would suggest or promote the use of more advanced and scientifically rigorous designs, using analytical epidemiologic case-control and prospective cohort studies. More than 50 studies, published since 2000, related the relationship between red meat consumption and colorectal cancer or prostate cancer, but do not support an independent and unequivocal association [33,34,35,36,37]. 

It is interesting to note that an ecologic study found no correlation between beef consumption and adjusted colorectal cancer incidence and mortality rates based on before 1970 USDA data [38]. Furthermore, no significant relationship was demonstrated between meat cooking methods, HCA or heme iron analyses, and prostate cancer [39]. The conclusions were that there was no observed increased in risk for advanced or lethal prostate cancer with ingestion of total red meat, fresh red meat, or processed meat, or any cooking method, HCA, or heme iron. 

## 5. Good and Bad Bacteria in Healthy Gut, Role of Iron

Disruptions to the human gut microbiome have been associated with pathological issues, metabolic diseases, cancer, and inflammatory bowel diseases. Also, issues in the gut microbiome can be a prelude to diabetes, obesity, and neurologic disorders. The human gut is estimated to contain over 10^14^ bacteria colonies or 10-fold the total number of cells in the human host. Certain gut bacteria such as *Lactobacillus*, maintain epithelial integrity, and likely regulate tight junction permeability, and prevent chemical-induced disruption, which can occur when bacterial toxins and proteins are triggered by inflammatory responses, characteristic of leaky gut syndrome or hyper intestinal permeability. Products from pathogenic commensal bacteria have been identified, such as short-chain fatty acids, which can disrupt the epithelial barrier integrity. 

**Figure 2:**
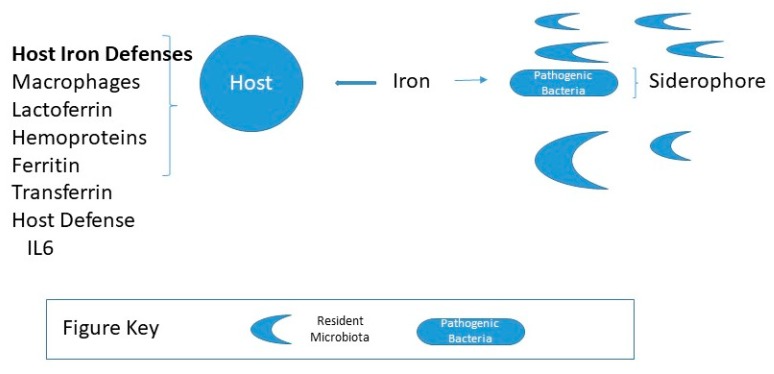
Host iron status and gut microbiota composition. For a healthy host, the amount of free iron is maintained from 10^−7^ to 10^−5^ M, insufficient concentrations for pathogenic bacteria to thrive predominantly if the microbiota is abundant in resident microbiota.Pathogenic bacteria, e.g., gram-negative, *Salmonella*, and *Shigella* produce iron-binding siderophores that enable them to scavenge iron [45].

**Figure 3:**
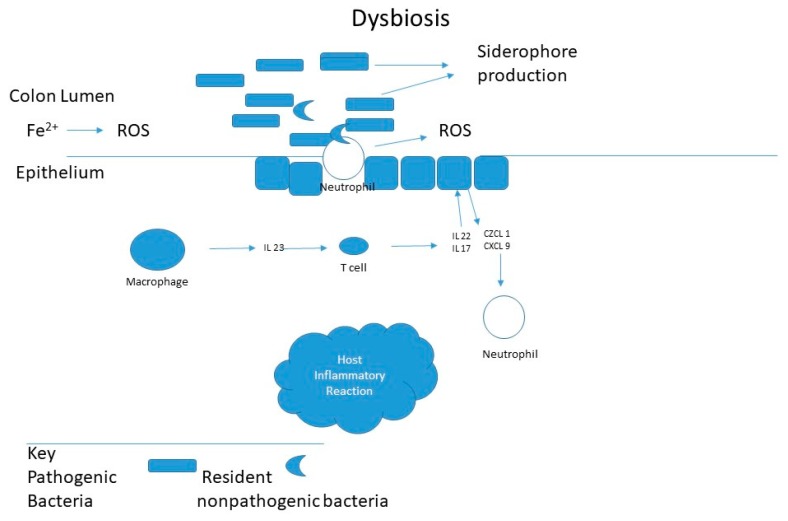
Implications of iron and oxidative stress in inflammatory gut and dysbiosis. The inflammatory response, triggered by intestinal pathogens, activates macrophages that stimulate T cells to secrete IL22 and IL17. Leading to the secretion of chemokines by the intestinal epithelium, which attracts neutrophils to the site of inflammation. Neutrophils that infiltrate will release reactive oxygen species (ROS) into the intestinal lumen, which can be augmented by lumen iron after dysbiosis [40].

Iron is an essential, growth-limiting nutrient for many gut bacteria, which along with the host, compete for dietary iron, illustrated in Figure 2. For gram-negative, *Salmonella* and *Shigella* or pathogenic forms *Escherichia coli*, iron acquisition plays an essential role in virulence and colonization. Such iron-requiring bacteria have iron-binding siderophores that enable them to absorb free iron or scavenge iron from hemoglobin or transferrin. *Lactobacilli* bacteria do not have siderophores and grow independently of iron. In contrast, *Lactobacilli*, a significant group of beneficial bacteria do not require iron and likely require manganese. As illustrated in Figure 3 it has been suggested that iron increases colorectal cancer by dysbiosis, causing a shift in the ratio of protective to pathogenic bacteria [40]. Specific bacteria have been implicated in carcinogenesis, such as *Streptococcus bovis, Bacteroides, Enterococcus faecalis,* and *Clostridia* [41,42,43,44].

In contrast, gut protective *Bifidobacterium longum* and *Lactobacillus acidophilus* may inhibit cancer by forming a protective barrier against colonization by pathogenic bacteria. *Bifidobacteriaceae* bind iron to their surface, reducing oxidative stress byproduct formation and bioavailable iron for pathogenic bacteria. *Lactobacillus* also appears to reduce the mutagenic effects of bile acids. 

In addition to a homeostatic role for iron [46], macrophages can contain microbial infections by depriving bacteria of iron. During infection and upon contact with mucous epithelia, bacteria encounter secretion of lactoferrin, also secreted by neutrophils recruited to the site of infection. These act to prevent bacteria from accessing iron for their own needs. 

Over the past few decades, the microorganisms residing in the gut have been recognized as a critical entity “organ” for human physiology and health. This organ may likely protect the host against pathogens and heighten metabolic competences. Several studies have reported on how the nutritional impact of animal and plant protein affects the intestinal microbial balance [47,48,49,50]. For example, incubation of cooked beef, chicken, or fish meat in vitro with human feces led to a difference in the population of *Bifidobacterium spp*. not—specifically *Bacteroides* [50]. In a rodent study, the intake of meat proteins increased the abundance of *Lactobacillus* in rat feces [51] compared to rats fed soy or casein protein, suggesting that an increase in such key players can protect the gut barrier against disruption by pathogens and reduce inflammation. As noted, excessive intake of red meat has been associated with a high risk of mortality from colorectal cancer and an unbalanced composition of the gut microbial population by high *Fusobacterium* and *Bacteroides* and low *Lactobacillus.* The difference in findings may be attributed to cooked meats, and lack of heme iron or the presence of heterocyclic nitrosation of peptide derived amines or nitrosylation reactions in the studies [51].

Iron fortification for infants is a concern [49]. Dietary iron can influence the composition of the infant in gut microbiota because of low absorption over 80% of the iron passing into the colon. Studies of iron fortification trials in infants have found an increased rate of diarrhea with adverse modifications of the gut microbiome and increased the abundance of pathogenic *Escherichia coli* and indications of intestinal inflammation. 

## 6. Significance of Dietary Antioxidants

Many studies have illustrated the relationship between fruit and vegetable protection against chronic diseases, a driving force for recommendations that half of your plate should be fruits and vegetables [52,53]. Fruits and vegetables are usually low in energy density and good sources of fiber, vitamins, and minerals. They are also usually good sources of a variety of phytochemicals, including polyphenolics and other bioactive substances. It is agreed that fruit and vegetables provide antioxidants and that plant-based foods introduce more antioxidants into human diets than non-plant foods [54] and likely defend against oxidative stress. Therefore, augmentation of the natural antioxidant defense systems through exogenous antioxidants provided by fruits and vegetables can favorably prevent or modulate such biological damage [8], including oxidative stress initiated by iron or heme iron entities. Likewise, the benefits of supplementing diets with polyphenolic nutraceuticals would be through the efficacious modulation of the antioxidant capacity of microbiota [55,56]. 

It is noteworthy that in addition to the usefulness of exogenous dietary antioxidants, endogenous compounds like bilirubin, which has antioxidant properties and hormonal functions [57,58], maybe a natural response of the body to rid heme levels. 

## 7. Impact of Dietary Antioxidants and Gut Bacteria 

In an in vitro study using enterobacteria to determine the effects of hemin and heating temperature on the mutagenicity and lipid oxidation of pork cooked with batters, researchers found increasing heating temperatures from 60 °C to 80 °C increased mutagenicity and lipid oxidation [56]. Also, the antioxidant activity of batter decreased with the increasing temperature; however, this was followed by an increase in antioxidant activity with mutagenicity gradually decreased as in vitro digestion continued. The author suggested increased antioxidant activities, and reduced mutagenicity was due to gut bacteria. Others have found that meat marinade could be a protective strategy to decrease colorectal cancer [59]. In contrast, studies where the gut may be infected with *Helicobacter pylori*, risk increases for inflammatory bowel diseases [44]. This suggests that antioxidant-rich foods may protect against heme-induced oxidation and promote healthy bacteria. Research is scant regarding if there is an inverse relationship between fruit and vegetable consumption vs. high red meat diets with various chronic diseases. One recent study demonstrated that high intakes of red meat were associated with a higher risk of all-cause and cardiovascular diseases (CVD) mortality [60]. In a study looking at red meat and flavonoid consumption, following a multivariable modification, there were no statistically significant relations between flavonoid intake and overall cancer risk in individuals with high levels of red meat intake. Such studies suggest that a high intake of meats is associated with poor health outcomes. Interestingly, men with low daily red meat consumption were shown to be inversely associated with flavonoid consumption and cancers [61]. 

## 8. Conclusion and Future Direction for Research 

Heme iron from meat remains an essential dietary constituent, mainly to prevent anemia. While multiple factors are likely operative, current mechanistic theories lack specificity for the exclusion of red meat iron. Chronic diseases may be catalyzed by iron initiated oxidative stress, and therefore, moderate consumption is still a useful preventative recommendation. Recommndations that include fruits and vegetables, and supplying rich sources of dietary antioxidants are judicious and mechanistically reasonable [55,60,61]. New research suggests that pathogenic bacteria, in the gut microbiome, play an important causative factor in chronic diseases, which in turn can be modulated by populating the gut with healthy bacteria. Promoting healthy microbiome bacteria can be encouraged by a diet combining fruits and vegetables rich in antioxidants and can be a useful strategy for the prevention of chronic diseases and possibly related to offset dietary heme iron. Likely, “you can have your meat and eat it too,” as long as fruits and vegetables are part of your healthy diet. Future work that focuses on the relationship (concentration-related effects) between diets containing heme iron and fruit and vegetable interactions would provide further insight and a better path for generating reasonable recommendations. 

## 9. Addendum 

Recently, four interlocking meta-analyses were published in the Annals of Internal Medicine [62,63,64,65]. Each study did a comprehensive sampling of the peer-reviewed literature using standard methods to survey the literature. In the first study, the conclusion was “the magnitude and adverse cardio metabolic outcome are very small, and the evidence is of low certainty.” The second study concluded, “the possible absolute effect of red and processed meat-eating on malignancy mortality and frequency are minimal, and the certainty of evidence is low to very low”, followed by the third study conclusion, “low or very low-certainty evidence suggested that dietary patterns with less red or processed meat-eating may result in minimal reductions in adverse cardio metabolic and cancer outcomes”. The final study noted that dietary choices have an active cultural component. Thus in such subjects, willingness to change meat consumption in response to health concerns is generally low and it is inappropriate to assume that informed persons would choose to reduce meat consumption based on small and distant health benefits, mainly if the benefits are uncertain. The overall recommendation was for adults 18 years plus, continuing current unprocessed red meat and processed meat consumption.
